# GPRI Biocommentary: Mohammed Abd Ellatif Nassar

**DOI:** 10.1038/s41390-022-02177-7

**Published:** 2022-07-07

**Authors:** Mohammed Abd Ellatif Nassar

**Affiliations:** 1 Pediatric Department, Faculty of Medicine, Tanta University, Tanta, Gharbia Governorate Egypt; 2 Pediatric department, Faculty of Medicine, Tanta University, Tanta, Egypt


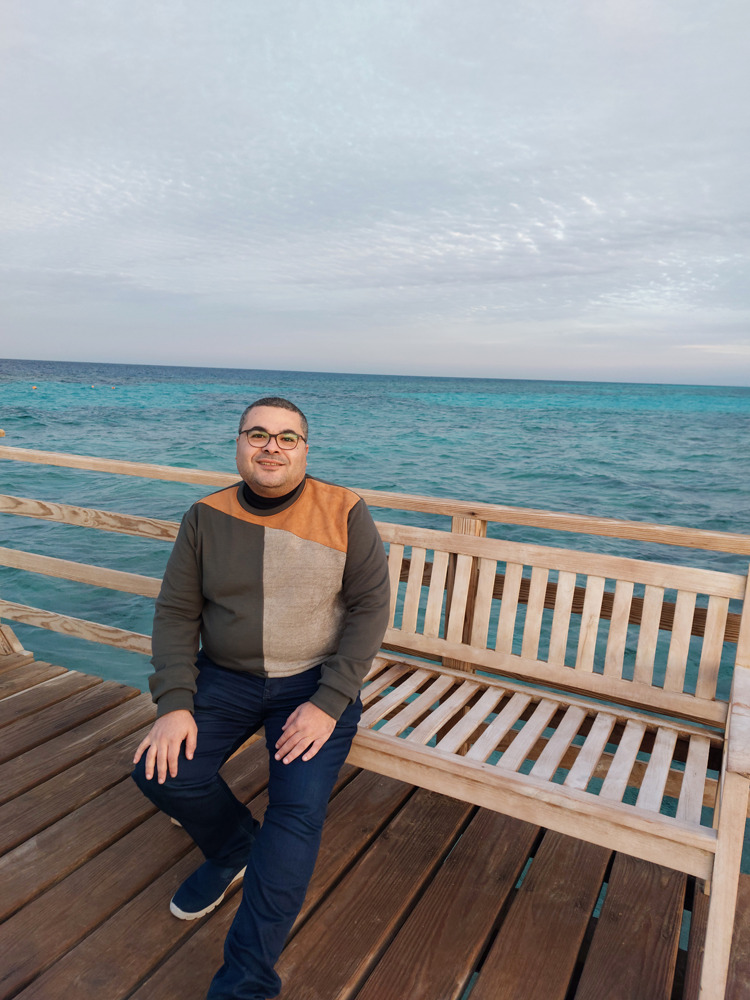


In 1980; I was born in Tanta, Egypt. In 2003, I graduated from the Faculty of Medicine, Tanta University with excellent grades and an honors degree and I was the thirteen in the medical school class and the first in the class in pediatrics. I spent a 1-year internship at Tanta University Hospital, and then I spent my pediatric residency for 3 years. I had my master’s degree of pediatrics in 2008. In 2015; I finished my MD and became a lecturer of Neonatology. Since my childhood I would like to be a doctor like my uncle Prof. Bayoumi Nassar professor of anesthesia and intensive care unit, but I had my decision to become a pediatrician during my study of pediatrics when I was a student in the Faculty of medicine. I had my interest in pediatric research after I finished my MD. My fields of interest are neonatology, pediatric cardiology and pediatric gastroenterology. I want to have new researches that can help children and neonates allover the world. I have cosupervised over 10 master’s and doctoral theses related to pediatrics. I have published more than 16 studies in several international and local journals. Prof. Mohammed Rewesha, Prof. Hamed Elsharkawy, Prof. Mostafa Awny, Prof. Abd ElRahman Elmashad, Prof. Heba El Mahdi and Prof. Doaa El Amrousy are my professors who helped me greatly in the field of pediatrics and neonatology. My parents had supported me with unlimited love and help all over my life. I am also grateful to my wife Dr Fatma Eldeeb, and my kids: Rana, Abd Ellatif, Ahmed and Lily for their patient, support and love. My advice to young researchers is to work hard, read more and more and try to have new ideas to add them to their field.

